# Taguchi Grey Relational Analysis for Multi-Response Optimization of Wear in Co-Continuous Composite

**DOI:** 10.3390/ma11091743

**Published:** 2018-09-16

**Authors:** Prasanth Achuthamenon Sylajakumari, Ramesh Ramakrishnasamy, Gopalakrishnan Palaniappan

**Affiliations:** 1 Department of Mechanical Engineering, PSG College of Technology, Coimbatore 641004, India; 2 Department of Mechanical Engineering, PSG Institute of Technology and Applied Research, Coimbatore641062, India; ramesh@psgitech.ac.in; 3 Department of Metallurgical Engineering, PSG College of Technology, Coimbatore 641004, India; pgk.metal@psgtech.ac.in

**Keywords:** Co-continuous composite, wear studies, multi-response optimization, Taguchi method, orthogonal array, ANOVA, grey relational analysis (GRA)

## Abstract

Co-continuous composites have potential in friction and braking applications due to their unique tribological characteristics. The present study involves Taguchi grey relational analysis-based optimization of wear parameters such as applied load, sliding speed and sliding distance, and their effect on dry sliding wear performance of AA6063/SiC co-continuous composite manufactured by gravity infiltration. A Taguchi L_9_ orthogonal array was designed and nine experimental runs were performed based on the designed experiments. The coefficient of wear and specific wear rate were recorded for each experiment. Based on the average responses computed from Taguchi grey relational analysis, an applied load of 60 N, sliding speed of 1 m/s and sliding distance of 1000 m were estimated to be the optimal parameters. An Analysis of Variance (ANOVA) was conducted to identify the predominant factor and established all the three factors as being significant. The sliding distance was found to have the highest significant influence of 61.05% on the wear of the C4 composite. Confirmation experiments conducted using the optimal parameters indicated an improvement of 35.25% in grey relational grade. Analysis of the worn surfaces of the confirmation experiment revealed adhesive and abrasive wear as the governing mechanisms.

## 1. Introduction

Ceramic/metal composites with interconnected and interpenetrating continuous network of a ceramic and metal phase are referred to as co-continuous ceramic composites (C4) [1]. The interlocking microstructure endows C4 with enhanced fracture toughness, superior wear resistance, higher stiffness and lower distortion with variation in operating temperature. This unique combination of desirable properties of C4 makes it suitable for applications entailing higher specific modulus, strength, creep resistance, corrosion resistance and improved wear characteristics [2,3]. Placing emphasis on weight reduction, numerous researchers have fabricated aluminum-based wear-resistant composite materials by reinforcing with high-strength and stiff ceramics such as B_4_C, SiC, Si_3_N_4_, TiB_2_ and TiC [4,5]. In C4 composites, the fraction of ceramic phase is greater than that of particulate reinforced composites. Consequently, they offer enhanced wear resistance [6,7]. Thus, three-dimensionally interconnected SiC porous preforms infused with Al alloys have potential in applications involving dry friction and wear [1,8]. 

Nong et al. [8] utilized SiC_3D_/Al co-continuous composites to prepare a ventilated shaft disc brake. The wear and friction performance of the C4 was found to be comparable to that of cast iron and steel. Moreover, better thermal conductivity and wear resistance was attained at half the density. Employing a digital logic approach, Maleque et al. [9] proposed Al-Cu alloys reinforced with 20% SiC particles as good candidate materials for brake discs. These composites possessed a higher friction coefficient and lower density compared with monolithic materials such as grey cast iron and Ti-6Al-4V alloy. A composite of AA6063/SiC was fabricated by Kamboj et al. [10] using the stir casting process and its mechanical properties were analyzed. An improvement of 47.3% in ultimate tensile strength (UTS), 50% in hardness and 60% in impact strength over the metal alloy were reported. This improvement in mechanical properties can be attributed to the presence of Mg as the principal constituent in AA6063, which acts as a surfactant and augments the wettability between Al and SiC by reducing the oxide layer on the surface of SiC [11]. Tribological properties of AA6063/clay composites have been explored by Agbeleye et al. [12] for brake disc rotor applications. It was reported that the sliding speed and applied load primarily influenced the wear rate. Though wear studies on AA6063/ceramic particulate composites have been reported, very few investigations on AA6063/SiC co-continuous composites exist. Therefore, to evaluate the propriety of AA6063/SiC C4 for friction and braking applications, wear studies are essential.

In order to investigate the multiple attributes associated with wear resistance of composites, numerous decision-making techniques, such as analytic hierarchy process (AHP), data envelopment analysis (DEA), grey relational analysis (GRA) and technique for order of preference by similarity to ideal solution (TOPSIS), have been suggested in literature [13]. Among them, grey relational analysis (GRA), proposed by Deng [14] in 1989, is one of the foremost techniques applied when the nature of information is incomplete and uncertain. Prayogo et al. [15] employed GRA in conjunction with a Taguchi orthogonal array to determine the optimal levels of multiple process parameters of EDM. Confirmation tests showed a significant increase of 29.44% in the grey relational grade indicating that GRA was effective in improving the performance characteristics of the EDM process. A combined Taguchi L_9_ orthogonal array and GRA approach has been adopted for optimization of injection molding parameters during fabrication of high-density polyethylene/TiO_2_ nanocomposites by Pervez et al. [16]. Using this approach, the optimal levels of operating variables were found to be holding time of 20 s, residence time of 30 min, TiO_2_ concentration of 5 wt.% and barrel temperature of 225 °C. Utilizing an L_12_ orthogonal array, Zou et al. [17] integrated S/N ratios of the Taguchi method with GRA to identify the significant contributors to surface treatment of concrete structures. The results indicated that the water-binder ratio with 89.99% and the weight of the pozzolanic material with 2.55% were the key factors influencing concrete protection. Multi-factor experiments of dry sliding wear in AA6531 metal matrix composites (MMCs) were performed by Uthayakumar et al. [18] using GRA and a Taguchi L_9_ orthogonal array. The analysis concluded that the applied load and sliding speed were the most influential factors. Additionally, load at 19.62 N and speed at 3 m/s were estimated as the optimal levels of factors. Ghosh et al. [19] evaluated the tribological performance of a composite comprising LM6 Al alloy with SiC_p_ of 37 µm mesh size using the Taguchi method and GRA. This study concluded that sliding time, with a contribution of 43.65%, significantly controlled the wear behavior of the composite. Furthermore, a 21% improvement of the grey relational grade was also realized thereby resulting in an improved design of the composite for tribological applications. In addition to this, the Taguchi method coupled with GRA has also been applied for multi-objective optimization of milling parameters [20,21], the turning process [22] and the wire electrical discharge machining process [23].

This study aims to optimize the dry sliding wear parameters to manufacture a desirable co-continuous ceramic composite with potential for friction and braking applications. Initially, a C4 of Al6063/SiC was fabricated using the gravity infiltration technique without the application of pressure [24]. Next, an experimental design with a Taguchi L_9_ (3^3^) orthogonal array was developed with three controlling factors, namely, the applied load, sliding speed and sliding distance. Three distinct levels were identified for each of these three parameters. Subsequently, nine experimental runs were conducted to obtain data for the multiple responses of the friction coefficient and specific wear rate. Thereafter, S/N ratios and GRA were utilized to analyze the responses in order to obtain the optimal parameters. The most significant factors affecting the wear behavior of C4 were then identified using ANOVA. Finally, using the optimal levels of design parameters, confirmation tests were conducted to verify the improvement of the quality characteristic, namely, the grey relational grade.

## 2. Materials and Methods 

### 2.1. Experimental Design

In order to evaluate the wear performance of the C4 specimen, three control factors, namely, the applied load (*L*, N), sliding speed (*S*, m/s) and sliding distance (*D*, m), each at three levels, were selected based on a literature review [6,25] as shown in Table 1. 

A Taguchi L_9_ orthogonal array (OA) [26] was designed with nine runs to conduct experiments. The wear performance was assessed using two response variables, namely, the coefficient of friction (COF), represented by µ, and the specific wear rate (*W*_s_). COF was computed as the ratio of the kinetic frictional force (*F*_f_) to the applied normal load (*F*_n_) as presented in Equation (1). *W*_s_ was calculated using Equation (2).

(1) $$\mathrm{Coefficient}\;\mathrm{of}\;\mathrm{Friction}\;(\mathrm{COF},\;µ)=\frac{\mathrm{Kinetic}\;\mathrm{frictional}\;\mathrm{force}\;(F_{f})}{\mathrm{Applied}\;\mathrm{normal}\;\mathrm{load}\;(F_{n})}$$

(2) $$\mathrm{Specific}\;\mathrm{Wear}\;\mathrm{Rate}\;(W_{s})=\frac{\Delta W}{\rho LD}\;({\mathrm{mm}}^{3}/\mathrm{Nm})$$

where Δ*W* is the weight loss of the pin (g), ρ represents the density of the specimen (g/mm^3^), *L* indicates the applied load (N) and *D* is the sliding distance (m). The complete experimental design of the OA along with the response values is discussed in Section 3. 

### 2.2. S/N Ratios in the Taguchi Method

The Taguchi method employs orthogonal arrays to reduce variance and optimize process parameters. In the Taguchi method, the signal to noise (S/N) ratio is used as a performance characteristic to measure process robustness and to evaluate deviation from desired values [27]. The S/N ratio, a logarithmic function, is computed by assessing the proportion of signal (mean) to the noise (standard deviation) [13]. In order to diminish noise and the effects of uncontrollable factors, higher values of S/N ratios are preferred [24]. High S/N ratios indicate improved quality of the product. There exist three types of S/N ratios, namely, higher-the-better, nominal-the-best and smaller-the-better as shown in Equations (3)–(5):

(3) $$\;{\left(\frac{S}{N}\right)}_{\mathrm{HTB}}=-10*\log_{10}\left(\frac{1}{n}\overset{n}{\underset{i=1}{\sum }}\frac{1}{y_{i}^{2}}\right)\;$$

(4) $$\;{\left(\frac{S}{N}\right)}_{\mathrm{NTB}}=10*\log_{10}\left(\frac{{\bar{y}}^{2}}{s^{2}}\right)\;$$

(5) $$\;{\left(\frac{S}{N}\right)}_{\mathrm{STB}}=-10*\log_{10}\left(\frac{1}{n}\overset{n}{\underset{i=1}{\sum }}y_{i}^{2}\right)\;$$

where n is the number of experiments, $y_{i}$ represents the response value of the *i*th experiment in the OA, ${\bar{y}}^{2}$ indicates the mean, and $s^{2}$ the variance of the observed data.

### 2.3. Multi-Response Optimization Using GRA

Taguchi’s experimental method is adequate to determine the optimal setting of process parameters for a single response characteristic. In the case of two or more responses, with dissimilar quality characteristics, multi-response optimization using GRA is the preferred method. Grey analysis can also be utilized to determine the similarity between seemingly irregular finite data [28]. Hence, multi-response optimization of wear parameters in this study is performed using the following steps in GRA.

#### 2.3.1. Grey-Relational Generation

In GRA, when the standard value and reference sequence range are considerably high, the function of the factors is neglected. Additionally, if the goals and directions of factors are disparate, GRA may yield inaccurate results. Hence, data pre-processing is performed to normalize the original reference sequences to a comparable sequence within the range of zero to one [29]. This approach of pre-processing data by normalization, into a group of sequences, is termed grey relational generation. In order to pre-process data using GRA, the response of the transformed sequences can be grouped into two quality characteristics, namely, larger-the-better or smaller-the-better. For smaller-the-better characteristic, the sequence can be normalized using Equation (6):

(6) $$\;x_{i}^{*}\left(k\right)=\frac{\max y_{i}\left(k\right)-y_{i}\left(k\right)}{\max y_{i}\left(k\right)-\min y_{i}\left(k\right)}\;$$

where $x_{i}^{*}\left(k\right)$ denotes the reference sequence after pre-processing for the ith experiment and $y_{i}\left(k\right)$ represents the initial sequence of the mean of the responses.

#### 2.3.2. Computation of Grey Relational Coefficient and Grade

Once the sequence is normalized, the next step is to calculate the deviation sequence of the reference sequence using Equation (7):

(7) $$\;\Delta_{0i}\left(k\right)=\left|x_{0}^{*}\left(k\right)-x_{i}^{*}\left(k\right)\right|\;$$

where $\Delta_{0i}\left(k\right)$, $x_{0}^{*}\left(k\right)$ and $x_{i}^{*}\left(k\right)$ refer to the deviation, reference and comparability sequences respectively. The grey relational coefficient (GRC) is then determined using Equation (8):

(8) $$\;\xi_{i}\left(k\right)=\frac{\Delta_{\min}+{\zeta \Delta }_{\max}}{\Delta_{0i}\left(k\right)+{\zeta \Delta }_{\max}}\;$$

where, $\xi_{i}\left(k\right)$ signifies the GRC of individual response variables computed as a function of $\Delta_{\min}$ and $\Delta_{\max}$, the minimum and maximum deviations of each response variable. The distinguishing or identification coefficient represented by ζ, defined in the range ζ ∈ [0,1], is generally set at 0.5 to allocate equal weights to every parameter. As shown in Equation (9), a composite grey relational grade (GRG), is then computed by averaging the GRC of each response variable:

(9) $$\;\gamma_{i}=\frac{1}{n}\overset{n}{\underset{i=1}{\sum }}\xi_{i}\left(k\right)\;$$

where $\gamma_{i}$ represents the value of GRG determined for the ith experiment, n being the aggregate count of performance characteristics. Once the optimal level of the factors is determined using GRG, the final step is to predict and verify the quality characteristics using Equation (10):

(10) $$\;\gamma_{\mathrm{predicted}}=\gamma_{m}+\overset{q}{\underset{i=1}{\sum }}\gamma_{o}-\gamma_{m}\;$$

where $\gamma_{o}$ denotes the maximum of average GRG at the optimal level of factors and $\gamma_{m}$ represents the mean GRG. The quantity q indicates the number of factors affecting response values. 

### 2.4. Analysis of Variance (ANOVA)

ANOVA is conventionally used to investigate whether the experimental design parameters have a significant effect on the responses. The ANOVA table is also widely used to analyze the interactions between factors and the effect of such interactions on the dependent variables [30]. Generally, the F-test is employed as a measure to evaluate the extent of factors controlling the test results. For a 95% confidence level, if the value of ‘Prob > F’, commonly known as ‘*p*-value’, is less than 0.05, the factors and interactions are considered significant [31]. Additionally, a large F-value is an indication of a process parameter having a significant effect on the performance characteristic. In ANOVA, the adjusted correlation coefficient, $R_{\mathrm{adj}}^{2},$ is used to evaluate the validity of the fitted model. $R_{\mathrm{adj}}^{2}$ measures the percentage of variation explained exclusively by those independent factors and interactions which predominantly affect the response variables. Further, to conclude that the created models fit the performed experiments well, it is desired that the values of $R^{2}$ and $R_{\mathrm{adj}}^{2}$ should be high and close to each other [30,31]. 

### 2.5. Materials and Specimens

**Figure 1:**
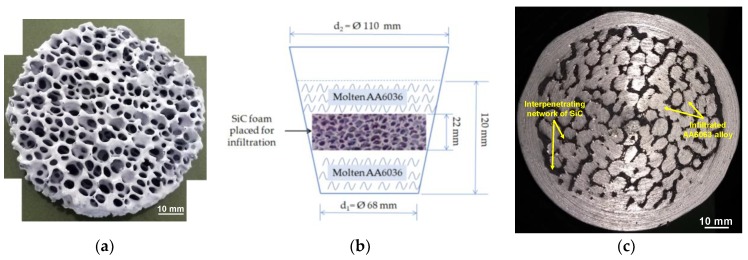
(**a**) SiC Foam of pore size 10 ppi; (**b**) the schematic of infiltration inside a clay graphite crucible; (**c**) the manufactured C4 composite showing the interpenetrating SiC and Al phases.

AA6063 commercial grade alloy and open cell SiC foam of diameter 80 mm, thickness 22 mm and pore size 10 pores per inch (ppi), were selected as the metal and ceramic phase, respectively. Compositional evaluation of AA6063 performed using SPECTROMAXx LMF04 (Kleve, Germany) optical emission spectrometer is presented in Table 2. The infiltration of AA6063 in SiC foam was performed in an electric resistance furnace capable of a maximum temperature of 1100 °C. The alloy was melted and superheated to 800 °C prior to pouring into another crucible containing SiC foam which was maintained at 800 °C to accelerate the kinetics of gravity infiltration [32]. The composite melt was maintained at this temperature for 3 h to ensure complete infiltration and the furnace chamber was purged with argon inert gas to avoid any atmospheric interaction. Subsequently, the infiltrated composite was allowed to solidify by furnace cooling. The co-continuous composite thus fabricated was machined out by a series of shaping, milling and grinding operations. Figure 1a shows the selected foam and Figure 1b depicts the scheme of infiltration of AA6063 in the SiC foam. Figure 1c exhibits the fabricated C4. The dark phases represent SiC network and shiny phases represent the Al alloy infiltrated to form the interpenetrating co-continuous structure. Using the Archimedes principle, the density of the composite was estimated to be 2.91 g/cm^3^.

**Figure 2:**
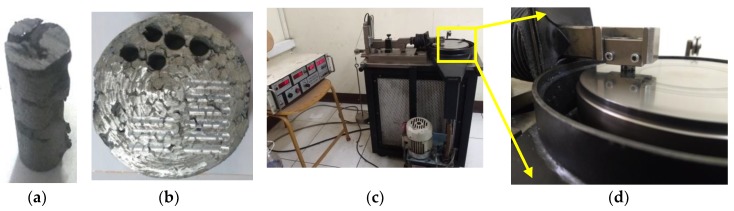
(**a**) The C4 wear test specimen extracted using abrasive water jet machine (AWJM); (**b**) the C4 composite after the AWJM process; (**c**) pin-on-disc tribometer; (**d**) the wear specimen mounted on the pin-on-disc machine.

To conduct tribological tests on the C4 composite, wear test specimens of diameter 13 mm were extracted using a three-axis CNC controlled Aquajet G3020 abrasive water jet machine (AWJM). The nozzle diameter and feed rate of the water jet were set at 1.1 mm and 80 mm/min respectively. Silica sand with a mesh number of 80 was used as an abrasive material for AWJM cutting. The wear test specimen extracted using the AWJM process is displayed in Figure 2a. Figure 2b depicts the C4 after the wear specimens have been extracted. The friction and dry sliding wear response of the co-continuous composite were evaluated using a Ducom TR20M36 (Ducom Instruments (Asia), Bangalore, India) pin-on-disc tribometer shown in Figure 2c. The tribometer is equipped with a data acquisition system which gathers and transmits information to WINDUCOM 2010 software (2010, Ducom Instruments (Asia), Bangalore, India) for processing and generating results. The specimens were subjected to sliding contact with an EN-32 steel disc of 65 Rockwell C hardness (HRC) under dry conditions as per ASTM G99-17 standards [33] as exhibited in Figure 2d.

## 3. Results and Discussion

In order to analyze Taguchi’s L_9_ OA using GRA, the experimental data in Table 3 were converted to S/N ratios. In this study, the effect of varying the control factors, *L, S* and *D* on the multiple responses of COF and *W*_s_ is analyzed. As lower values of COF and W_s_ are desirable, smaller-the-better quality characteristic was selected to investigate the influence of factors on the multiple responses. This section is subdivided into four parts. Section 3.1 and Section 3.2 report the analysis of S/N ratios to identify predominant factors. Section 3.3 and Section 3.4 explain the outcomes of GRA. Section 3.5 and Section 3.6 present the results of confirmation tests and interfacial characterization. Section 3.7 discusses the morphology of the worn surface. 

### 3.1. Effect of the Control Factors on COF

The experimental data of COF in Table 3 were converted into S/N ratios using Minitab^®^ 17 statistical software (Version 17, Minitab, Ltd, Coventry, UK). The dominant control factors were identified from the delta statistics in the response table for S/N ratios as shown in Table 4. The delta statistics were computed based on the difference between the highest and the lowest average value of each factor. Ranks were then assigned according to the delta value. The highest value of delta was assigned the first rank and represents the predominant factor affecting COF. Table 4 indicates that the applied load with a delta value of 2.146 is the most influential factor. The second most contributing factor is the sliding speed with a delta value of 0.568, followed by the sliding distance of 0.181.

**Figure 3:**
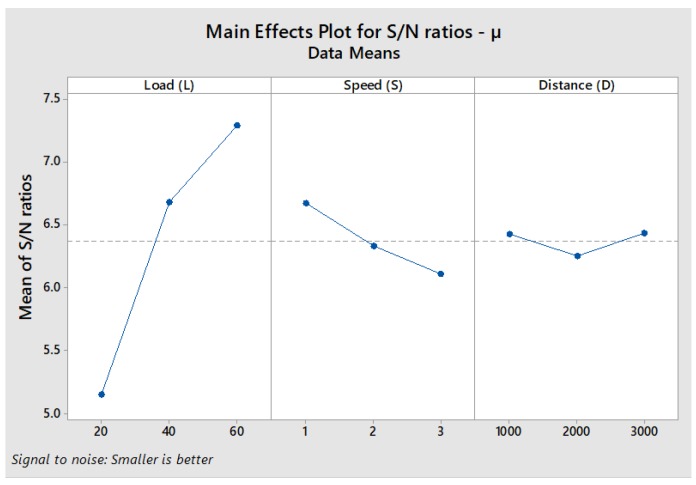
Main Effects Plot for S/N Ratios of µ.

From the responses in Table 4, the main effects plot for S/N ratios was generated as depicted in Figure 3. The trend of the plot indicates that COF is greatly influenced by variations in the applied load. As can be deciphered from Table 3, particularly in the case of Al-SiC composites, the friction coefficient normally decreases with an increase in the applied load. A possible reason is the formation of a tribo-layer at high loads [34]. The steeply increasing trend of S/N ratios for COF from 20 N to 60 N in Figure 3 supplements the observation that the quality of response is improved when the applied load increases. The relationship between sliding speed and S/N ratio in Figure 3 shows a decreasing trend for speeds from 1 m/s to 3 m/s. The plot of sliding distance shows a decrease of S/N ratio from 1000 m to 2000 m. Subsequently, the value considerably increases when the sliding distance is increased to 3000 m. In S/N ratio analysis, regardless of the quality characteristic, a higher S/N ratio corresponds to better values of experimental results; in this instance, a lower COF [34]. The response table presented in Table 4 and main effects plot for S/N ratios in Figure 3 suggest that L3, S1 and D3 are the desired factor levels in order to achieve high S/N ratios and lower values of COF.

Subsequent to determining factor levels, an ANOVA (shown in Table 5) was performed to obtain the percentage contribution of each factor’s effect on the COF. It can be observed that the applied load, with a contribution of 92.66%, has the highest influence on the COF followed by sliding speed, with 6.21%, and then sliding distance, with 0.82%. At 95% confidence level, factors having a *p*-value less than 0.05 are considered significant [35]. Since the *p*-values of applied load and sliding speed are less than 0.05, they are considered significant contributors to the change in COF. Further, it can be seen from Table 5 that the values of R^2^ and R^2^_adj_ are high and comparable with each other. This indicates the goodness of fit of the model [36].

### 3.2. Effect of the Control Factors on W_s_

In order to determine the factors influencing the specific wear rate, the S/N ratios of the experimental data of *W*_s_ were calculated as shown in Table 3. Similar to COF analysis, smaller-the-better characteristic of the Taguchi method was selected to investigate factor effects. The response table for the S/N ratios of *W*_s_ was then generated as shown in Table 6. The results indicate that the applied load, with a delta of 3.6, has the highest effect on *W*_s,_ followed by sliding distance and sliding speed, with deltas of 0.766 and 0.68, respectively. 

**Figure 4:**
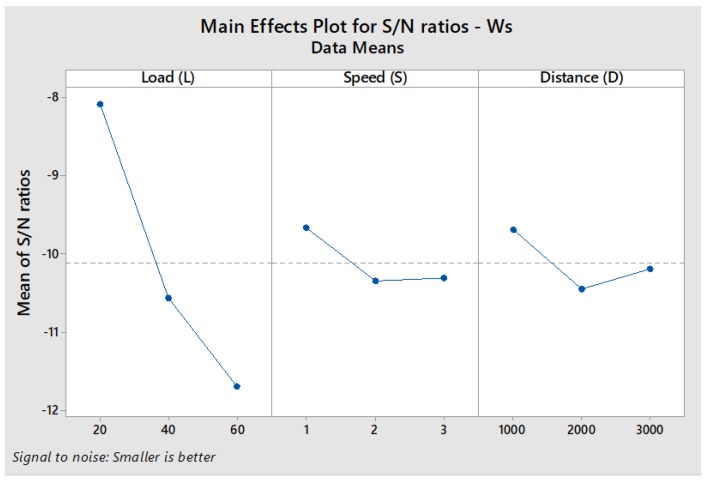
Main Effects Plot for S/N Ratios of *W*_s._

The response table for S/N ratios was then used to obtain the plot of main effects for *W*_s,_ as exhibited in Figure 4. It can be deciphered that the ratio decreases with an increase in the applied load and varies marginally with sliding speed. In the case of sliding distance, there is a slight decrease in the ratio between 1000 m and 2000 m and then an appreciable increase until 3000 m. Figure 4 shows that desirable values of S/N ratios of *W*_s_ are achieved at the first level of applied load (L1), the first level of sliding speed (S1) and the first level of sliding distance (D1). The results of S/N ratios in Table 6 also specify the same levels for the variables. Further, the delta statistics in Table 6 reveal that *W*_s_ is primarily affected by applied load, followed by sliding distance and sliding speed. 

Thereafter, to determine the significant factors and the percentage contribution of each factor to the response *W*_s_, an ANOVA was performed (Table 7). It can be inferred from Table 7 that, at the 95% confidence level, the applied load has the highest percentage contribution of 80.3%, followed by sliding distance of 16.79%. Further, as the *p* value of the applied load is less than 0.05, it is the sole significant factor. The high *R* values indicate that 91.78% of the variability can be explained by the model and hence, affirms the validity of this model.

The S/N ratio approach detailed above generates two sets of optimal input parameters for each of the two responses. However, in real-world applications, it is essential to determine a single combination of optimal parameters for multiple responses [37]. Hence, in this study, GRA is deployed for multi-response optimization of the three factors and two responses. 

### 3.3. Multi-Response Optimization Using GRA

GRA is primarily employed to solve practical problems comprising a limited set of data. It is typically utilized to approximate the behavior of uncertain systems with no black and white solution. In the grey system, black implies having no information and white signifies having all information [14]. GRA has been widely applied to optimize problems involving multiple factors and responses [17,37,38]. Data pre-processing by grey relational generation was performed on the experimental data of the responses in Table 3, namely, COF and *W*_s_. The reference sequence of the responses in the range of 0 to 1 was obtained by normalizing the data using Equation (6). Subsequently, the deviation sequences were computed using Equation (7). Table 8 displays the reference and deviation sequences obtained after data pre-processing.

**Figure 5:**
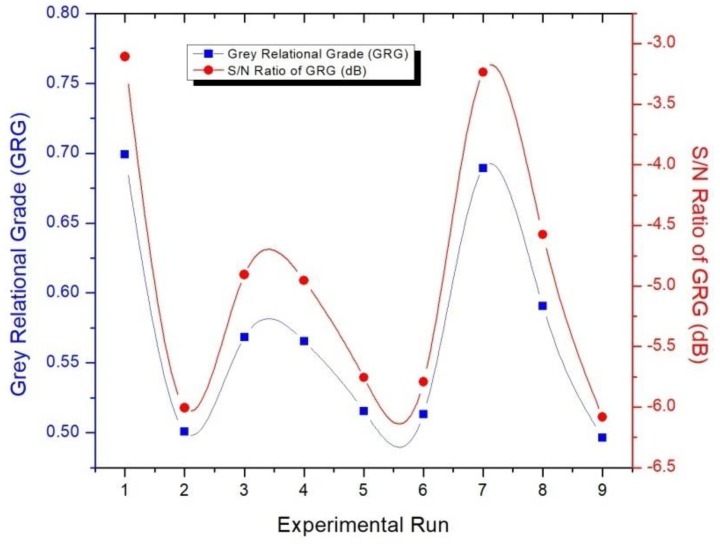
Plot of GRG vs S/N Ratios.

Once the deviation sequences were ascertained, the GRC ($\xi_{i}\left(k\right))$ for each value of the response was calculated using Equation (8). Finally, the average of the GRCs was computed to determine the GRG ($\gamma_{i}$). As listed in Table 9, the computed values of GRGs were utilized to generate the corresponding S/N ratios. A higher value of S/N ratio is beneficial and indicates that the experimental data lies close to the ideal normalized value of GRG [37]. Figure 5 exhibits the plot of GRG vs. S/N ratio. It shows that the first experimental run has the highest S/N ratio. Accordingly, the first rank was assigned to the first experimental run. The trailing nature of the GRG, beneath the plot of S/N ratios in Figure 5, also supplements the discussion above.

Once the ranks were ascertained, a response table for the GRG was designed. The GRG of each factor at the chosen level was selected and averaged to generate the mean of GRG for individual factors. For instance, the parameter applied load was set at level 2 in the fourth, fifth and sixth runs of the experiment. The corresponding GRG values from Table 9 were utilized for calculation as shown in Equation (11).

The mean of selected GRGs was calculated using the procedure above and assembled to create the response table shown in Table 10. The grades in the response table serve as a measure of the correlation between the reference sequence and comparability sequence of GRA. Higher values of the mean of GRGs indicate a strong correlation [38]. Therefore, from the response table of GRGs in Table 10, it is possible to arrive at the combination of optimal parameters which maximize overall response. From Table 10, the maximum grey relational grades exist at L3, S1 and D1. Hence, to conclude, the optimal settings for beneficial wear behavior of C4 composite are the applied load at 60 N, sliding speed at 1 m/s and sliding distance at 1000 m.

(11) $$L\;(\mathrm{level}\;2)=\frac{0.5654+0.5156+0.5134}{3}=0.5314$$

### 3.4. ANOVA for GRG

In order to investigate the significance and percentage contribution of each factor on the multiple wear performance characteristics of C4, an ANOVA was performed for the grey relational grade at a 95% confidence level. Considering the multiple responses of COF and *W*_s_, Table 11 shows that the sliding speed has the highest effect, of 61.04%, on the GRG, followed by the sliding distance, with 24%, and applied load, with a minimal influence of 14.80%. Further, since the *p*-values of all factors are less than 0.05, it can be inferred that all parameters significantly influence the wear performance of the C4. The high R values signify the goodness of fit of the developed model.

### 3.5. Confirmation Tests

Once the optimal settings have been identified, the final step in grey relational analysis is to predict and verify the performance improvement of the responses. The predicted GRG was computed using Equation (10). Confirmation tests were conducted to validate the outcomes of the analysis and the average GRG of three runs was calculated. For the optimized conditions, the coefficient of wear and wear rate was found to be 0.428 and 3.56 respectively. Further, it can be inferred from Table 12 that the results of the confirmation experiment are in good agreement with the predicted values. Additionally, an improvement of 35.25% in GRG is also obtained. This improvement in the experimental results over the initial design parameters affirms the validity of the Taguchi method coupled with grey relational analysis for enhancing the wear performance of C4.

### 3.6. Interfacial Characterization of Infiltrated C4 

**Figure 6:**
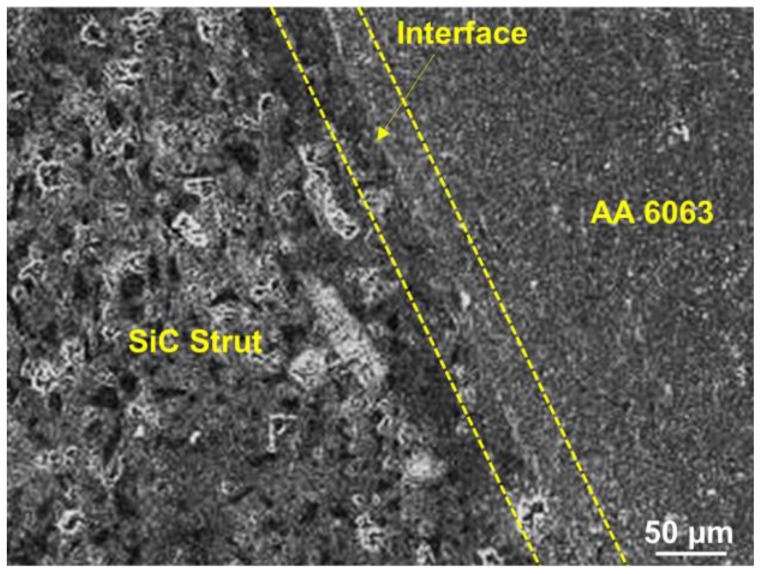
SEM Micrograph of AA 6063/SiC interface.

The micrograph obtained using a Carl Zeiss EVO 18 scanning electron microscope (SEM, Oberkochen, Germany) shown in Figure 6 indicates that no evident pits or cracks can be observed along the interface in the infiltrated C4. Additionally, no interfacial product was observed within the detection limit of SEM analysis, thereby indicating that the infiltration is effective and good bonding exists between the metal and ceramic phases of the C4.

### 3.7. Worn Surface Morphology

**Figure 7:**
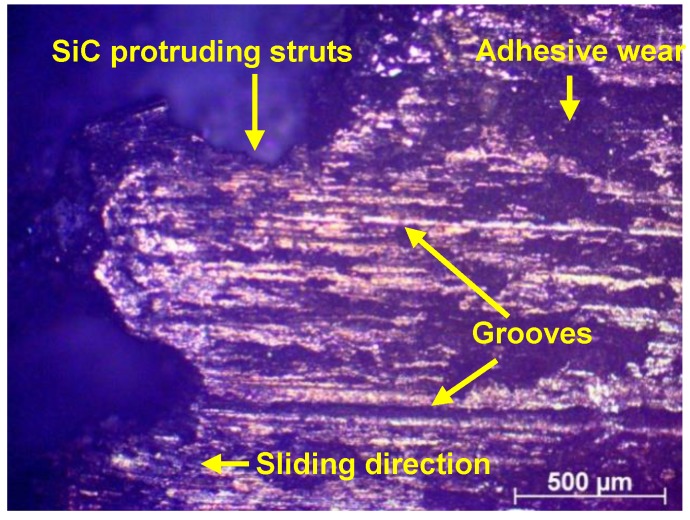
Optical micrograph of C4 composite exhibiting wear tracks at the Al-SiC interface.

A Carl Zeiss Axio Scope A1 (Oberkochen, Germany) polarized light microscope was used to obtain an optical micrograph of the worn surface of the C4 sample at the optimal design parameters. The micrograph in Figure 7 exhibits a distinct groove and ridge wear pattern on the Al phase, parallel to the direction of sliding. This pattern is due to micro-ploughing caused by the steel counter-face of the tribometer on the soft metal. The observations above agree well with reported literature [12,39]. In addition to the grooves along the sliding direction, the presence of a prow and pit pattern suggests the occurrence of minor adhesive wear on the Al phase along with predominantly abrasive wear. Prior studies [25] on the wear mechanism of C4 composites have shown evidence of SiC ceramic struts protruding from the surface after initial wear. This phenomenon can also be observed in Figure 7. Further research on the improvement of wear resistance of C4 can be conducted by varying the metal-ceramic phases, processing conditions and pore-size of the ceramic foam.

**Figure 8:**
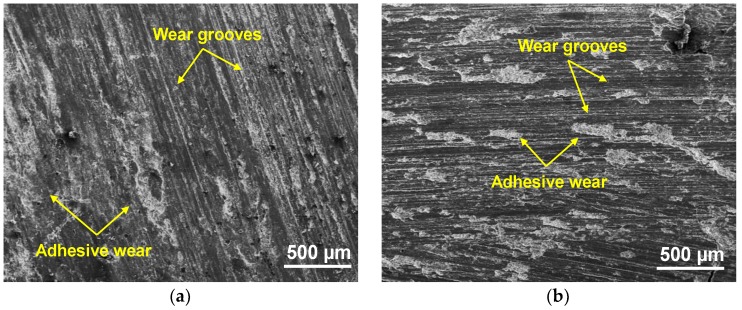
SEM micrograph of worn surface of C4 at: (**a**) initial design levels; (**b**) optimal design levels.

In order to gain additional insights on the wear mechanism and detect elemental composition at the AA6063/SiC interface of the C4, wear specimens at the initial and optimal design parameters were examined with a Carl Zeiss EVO 18 SEM equipped with EDAX energy dispersive spectrometer. The SEM micrograph at initial design parameters (L2, S3, D1) in Figure 8a, clearly shows the grooves along the sliding direction. At moderate loads (L2) and high speeds (S3), heat generated due to friction between the rotating pairs—namely, the steel disc and C4 specimen—results in softening of the worn surface. In particular, the grains of the Al phase tear and adhere to the hard surface of SiC. This causes smearing of Al across the surface of SiC resulting in an uneven adhesive wear combined with abrasive wear as shown in Figure 8a. In the case of wear of C4 at optimal levels (L3, S1, D1), the wear tracks in Figure 8b indicate that the magnitude of abrasive wear has considerably diminished. This may be attributed to the occurrence of steady-state wear due to the premature exposure of SiC foam struts at high load (L3) and slow speed (S1) [25] resulting in uniform adhesive wear along with predominant abrasive wear at optimal levels.

**Figure 9:**
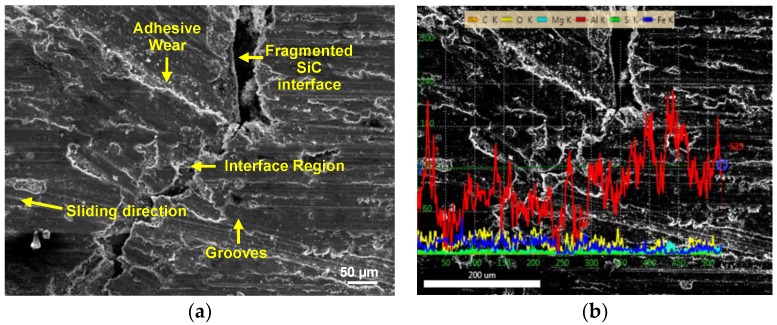
Worn surface morphology at the AA6063/SiC interface: (**a**) SEM micrograph; (**b**) line scanning elemental analysis.

**Figure 10:**
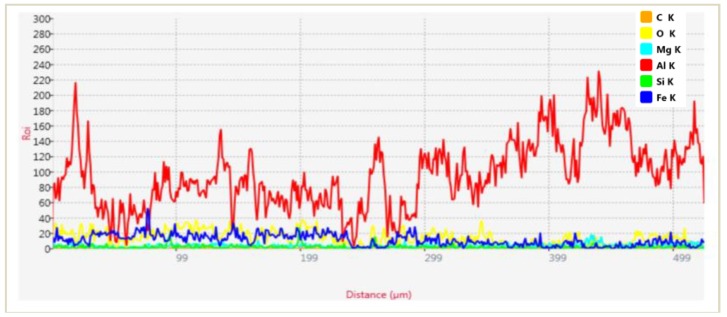
EDAX analysis of the interface.

Figure 9 and Figure 10 show the elemental mapping obtained by EDAX analysis of interface. The map of distance vs region of interest (Roi) shows an enhanced presence of O and Fe between 50 µm and 200 µm (interface region) in addition to the constituents of AA6063 (Al-Mg-Si). This suggests that the particles of Fe from the steel counter-face of the disc in the tribometer may have embedded on the C4. Additionally, the contact of the steel disc with the wear specimen under high load (L3) in ambient air results in passive oxidation of SiC [6]. This is confirmed by the elevated presence of O in the line scanning analysis of the interfacial zone in Figure 10. When subjected to further wear, the brittle oxide layer disintegrates, forming a crack along the interface as shown in Figure 9a.

## 4. Conclusions

The primary aim of this study was to obtain the optimal set of parameters which affect the wear performance of a co-continuous ceramic composite in the presence of multiple responses. Initially, the outcome of varying three factors—namely, applied load, sliding speed and sliding distance—on the multiple responses of the coefficient of friction and wear rate was studied using a Taguchi L_9_ OA and GRA approach. From the response table of the grey relational grades, the optimal set of parameters for enhanced wear performance of the C4 composite were identified to be an applied load of 60 N, a sliding speed of 1 m/s and a sliding distance of 1000 m. The ANOVA for GRG indicated that the *p* values of all parameters were less than 0.05 and, hence, significant. Finally, confirmation tests were performed to verify the improvement of 35.25% in GRG, from 0.5134 for the initial design parameters (L2, S3, D1), to 0.6944 for the optimal parameters (L3, S1, D1). SEM analysis of the worn surface revealed steady-state wear of C4 at optimal setting of parameters. 

## Figures and Tables

**Table 1:** Control factors of wear and their levels.

Control Factors	Unit	Symbol	Levels		
			1	2	3
Applied load	N	*L*	20	40 ^1^	60
Sliding speed	m/s	*S*	1	2	3 ^1^
Sliding distance	m	*D*	1000 ^1^	2000	3000

^1^ Initial testing level.

**Table 2:** Chemical composition of AA6063.

Element	Al	Mg ^1^	Si ^1^	Fe	Cr	Cu
wt.%	98.46	0.642	0.387	0.210	0.034	0.026

^1^ Principal alloying element.

**Table 3:** Taguchi L_9_ OA and multi-response results with signal to noise (S/N) Ratio.

Run	Control Factors			Response Values		S/N Ratio (dB)	
	*L* (N)	*S* (m/s)	*D* (m)	µ	*W*_s_ × 10^−3^ (mm^3^/Nm)	µ	*W*_s_
1	20	1	1000	0.531	2.31	5.4981	−7.2722
2	20	2	2000	0.559	2.79	5.0518	−8.9121
3	20	3	3000	0.57	2.53	4.8825	−8.0624
4	40	1	2000	0.457	3.22	6.8017	−10.1571
5	40	2	3000	0.463	3.52	6.6884	−10.9309
6 ^1^	40	3	1000	0.471	3.38	6.5396	−10.5783
7	60	1	3000	0.411	3.79	7.7232	−11.5728
8	60	2	1000	0.434	3.63	7.2502	−11.1981
9	60	3	2000	0.452	4.11	6.8972	−12.2768

^1^ Initial design run.

**Table 4:** Response table for S/N ratios of µ.

Level	L	S	D
1	5.144	**6.674** ^1^	6.429
2	6.677	6.33	6.25
3	**7.29** ^1^	6.106	**6.431** ^1^
Delta	2.146	0.568	0.181
Rank	1	2	3

^1^ Desired factor levels.

**Table 5:** ANOVA for S/N ratio of µ.

Source	DF	Adj SS	Adj MS	*F*	*P*	Contribution (%)	Remarks
*L*	2	7.3305	3.6653	299.80	0.003	92.66	significant
*S*	2	0.4910	0.2455	20.08	0.047	6.21	significant
*D*	2	0.0649	0.0324	2.65	0.274	0.82	not significant
Error	2	0.0245	0.01223			0.31	
Total	8	7.9108		*S* = 0.11057		R^2^ = 99.69%	R^2^_adj_ = 98.76%

**Table 6:** Response table for S/N ratios of *W*_s_.

Level	*L*	*S*	*D*
1	**−8.082**	**−9.667**	**−9.683**
2	−10.555	−10.347	−10.449
3	−11.683	−10.306	−10.189
Delta	3.6	0.68	0.766
Rank	1	3	2

**Table 7:** ANOVA for S/N ratio of *Ws*.

Source	DF	Adj SS	Adj MS	*F*	*P*	Contribution (%)	Remarks
*L*	2	20.3496	10.1748	43.85	0.022	90.06	significant
*S*	2	0.8713	0.4356	1.88	0.348	3.86	not significant
*D*	2	0.9098	0.4549	1.96	0.338	4.03	not significant
Error	2	0.4640	0.2320			2.05	
Total	8	22.5947		*S* = 0.481689		R^2^ = 97.95%	R^2^_adj_ = 91.78%

**Table 8:** Reference and deviation sequences after data pre-processing.

Run	Reference Sequences, $x_{i}^{*}$		Deviation Sequences, $\Delta_{0i}$	
	µ	*W*_s_	µ	*W*_s_
1	0.2453	1	0.7547	0
2	0.0692	0.7333	0.9308	0.2667
3	0.0000	0.8778	1.0000	0.1222
4	0.7107	0.4944	0.2893	0.5056
5	0.6730	0.3278	0.3270	0.6722
6	0.6226	0.4056	0.3774	0.5944
7	1.0000	0.1778	0.0000	0.8222
8	0.8553	0.2667	0.1447	0.7333
9	0.7421	0	0.2579	1

**Table 9:** Rank of grey relational grade (GRG) with S/N ratios.

Run	Grey Relational Co-Efficient ξ_i_ (k)		Grey Relational Grade γ _i_	S/N Ratio of GRG	Rank
	µ	*W*_s_			
1	0.3985	1	0.6992	−3.1074	1
2	0.3495	0.6522	0.5008	−6.0065	8
3	0.3333	0.8036	0.5685	−4.9061	4
4	0.6335	0.4972	0.5654	−4.9536	5
5	0.6046	0.4265	0.5156	−5.7546	6
6	0.5699	0.4569	0.5134	−5.7913	7
7	1.0000	0.3782	0.6891	−3.2347	2
8	0.7756	0.4054	0.5905	−4.5755	3
9	0.6598	0.3333	0.4965	−6.0809	9

**Table 10:** Response table of GRGs.

Factors	Level 1	Level 2	Level 3	Delta	Rank
L	0.5895	0.5314	**0.5920**	0.0606	3
S	**0.6512**	0.5356	0.5261	0.1251	1
D	**0.6010**	0.5209	0.5910	0.0801	2

Mean of GRG = 0.5710.

**Table 11:** ANOVA for grey relational grade.

Source	DF	Adj SS	Adj MS	F	P	Contribution (%)	Remarks
L	2	0.007054	0.003527	93.82	0.011	14.80	significant
S	2	0.029105	0.014552	387.1	0.003	61.04	significant
D	2	0.01144	0.00572	152.16	0.007	24.00	significant
Error	2	0.000075	0.000038			0.16	
Total	8	0.047674		S = 0.0061314		R^2^ = 99.84%	R^2^_adj_ = 99.37%

**Table 12:** Results of the confirmation experiment.

Initial Design Parameters		Optimal Design Parameters	
		Prediction	Experiment
Setting level	L2 S3 D1	L3 S1 D1	L3 S1 D1
Grey relational grade	0.5134	0.7023	0.6944
Improvement in GRG		36.79%	35.25%
